# A novel olfactory sorting task

**DOI:** 10.1007/s00405-024-08811-w

**Published:** 2024-07-13

**Authors:** Shubin Li, Anne Wolter, Christine Kelly, Barry Smith, Katie Whitcroft, Harry Sherwood, Beth Longley, Thomas Hummel

**Affiliations:** 1 Department of Otorhinolaryngology, Smell & Taste Clinic, Technische Universität Dresden, Dresden, Germany; 2 AbScent, 14 London Street, Andover, Hampshire, UK; 3 Institute of Philosophy, School of Advanced Study, University of London, London, UK; 4 Centre for Olfactory Research and Applications, School of Advanced Study, University of London, London, UK; 5 Institute of Cognitive Neuroscience, UCL, London, UK

**Keywords:** Odor memory, Self-administered test, Memory match game, Olfactory test, Early diagnosis of olfactory dysfunction

## Abstract

**Background:**

This study aimed to develop a simple self-administered screening tool for odor memory, which allowed users to self-test their olfactory function repeatedly even at home.

**Methods:**

One hundred and ten participants were recruited (30 men, age = 50.1 ± 9.9 years; 80 women, age = 47.1 ± 11.5 years); half of them were heathy volunteers, the other half were patients with olfactory dysfunction. Fifty-one healthy participants volunteered for a retest within an interval of a maximum of 14 days. Olfactory function was assessed using the extended Sniffin’ Sticks test (SST) comprising tests for odor threshold, identification, and discrimination. All participants received the Novel Olfactory Sorting Task (NOST) which is based on the sorting of 12 matching pairs of odors involving olfactory and cognitive functions. After that, all participants rated questions related to their test performance and the practicability of the test.

**Results:**

Consistent with the previous literature, significant effects of age were found. Results showed an acceptable test-retest reliability and a satisfactory validity of the NOST. The NOST score not only had positive correlations with SST, but also was capable of differentiating severe hyposmia/anosmia from normosmia by the score of 5.5 (sensitivity of 76.2%, specificity of 77.6%).

**Conclusion:**

The present study showed the good reliability, validity, and possible clinical usefulness of the NOST. As a self-performed screening test, it can be comprehended and conducted easily, which may provide a quick and simple approach to obtaining a global estimation of olfactory and cognitive functions.

**Supplementary Information:**

The online version contains supplementary material available at 10.1007/s00405-024-08811-w.

## Introduction

Based on previous literature, the prevalence of olfactory dysfunction varies from 1.4 to 29% [1, 2]. The COVID-19 pandemic has further increased this proportion to some degree [3]. However, it has been observed that many individuals were unaware of their olfactory dysfunction. Surveys such as that conducted by Wehling et al., [4] have shown the proportion of olfactory dysfunction unawareness was 86% in middle-aged participants and 78% in older people. Some people with congenital anosmia may not even realize the absence of the sense of smell for most of their lifes [5, 6]. Although the COVID-19 pandemic has brought widespread awareness of olfactory dysfunction [7], many people only notice an olfactory loss when olfaction is severely or even totally impaired. Unlike meta-cognitive awareness of our vision or audition, our self-rated olfactory function or dysfunction is not reliable and poorly correlated with valid and reliable psychophysical tests. People regularly report having a poor sense of smell despite performing well with respect to psychophysical olfactory tests. In contrast, people claiming to have a sensitive sense of smell often have average or poor scores when tested [8].

Currently, there are few tests available that would allow people to quantitatively and repeatedly examine their sense of smell accurately in a home environment. Li and his colleagues [9] introduced a chemosensory home test including tests for smell, taste, and trigeminal functions, but no odor memory tests were included. Therefore, the development of simple odor memory tests allowing for self-administration appears to be of importance.

Odor memory comprises a complex and advanced cognitive function [10]. The successful completion of an odor recognition memory task not only requires odor detection, discrimination and naming, but also requires the subject to encode, store and retrieve odor information [10]. Therefore, odor recognition memory could be a comprehensive indicator to evaluate one’s olfactory function in general.

Several well-validated, easy and quick tests are available to examine odor memory. For example, the Sniffin’ TOM [11] asks participants to identify 8 targets from 16 odors, or 16 targets from 32 odors in an extended version (TOM-32) [12]; the Olfactory Memory Test Battery (OMTB) uses delayed matching-to-sample and n-back paradigms to assess odor recognition and working memory separately [13]; the Odor Memory Test (OMT) is conducted in a forced-choice procedure with 4 odors repeatedly used in 12 trials, requiring participants to identify (microencapsulated) odors [14]. Despite of the contributions that these tests have made in clinical diagnosis and scientific research, such approaches are of limited value because they are designed as screening tests or because they are difficult to self-administer.

The aim of the present study was to investigate a self-administered olfactory memory test (the Novel Olfactory Sorting Task, NOST) based on a prototype designed by one of the authors as a game that serves as an odor matching memory task, that would allow individuals to repeatedly self-test their olfactory function.

## Method

### Participants and materials

One hundred and ten participants were recruited (30 men, age = 50.1 ± 9.9 years; 80 women, age = 47.1 ± 11.5 years) for the study; half of them were heathy volunteers, the other half were patients with olfactory dysfunction who presented themselves to the Smell & Taste Clinic, Department of Otorhinolaryngology, Technical University of Dresden (Table 1). Fifty-one healthy participants volunteered for a retest within an interval of a maximum of 14 days. The study design had been approved by the ethics committee at the University Clinic of the Technische Universität Dresden (application number EK378082019). All participants provided written informed consent.

**Table 1:** Twelve components of NOST

Number	Fragrance	CAS Number	Quality
**1**	Rosemarel	6931-54-0	eucalyptus, camphor
**2**	Orivone	16587-71-6	woody, earthy
**3**	Citral Dimethyl Acetal	7549-37-3	lemony
**4**	Anisyl Acetate	104-21-2	Sweet, fruity, floral
**5**	Allyl-Cyclohexyl Propionate	2705-87-5	Fruity, pineapple, sour
**6**	Cumin Nitrile	13816-33-6	Spicy, powdery
**7**	Helvetolide	141773-73-1	Musk
**8**	Kephalis	36306-87-3	Woody, amber
**9**	Undecavertol	81782-77-6	Watery, floral, green
**10**	Isocyclocitral	1335-66-6	Green, grassy, ivy
**11**	Magnolan	27606-09-3	Floral, rose
**12**	Ethyl Maltol	4940-11-8	Food, sweet, delicious, cotton candy

Note: “Helvetolide” is a trademark of Firmenich SA and it can be found in https://www.startupwala.com/trademarks-registration/search-DELHI-HELVETOLIDE-1484177

The final set of 12 odors was selected through 4 rounds of evaluations by 3 experts from a pool of 36 odors (see supplementary). These odorants were evaluated on several dimensions in a 0–10 scale: chemical complexity, valence, familiarity, chemical stability, and perceptual stability. First, we excluded odorants which did not possess a single-molecule structure. Second, the evaluation of valence excluded unpleasant odorants scoring below 4 to ensure a favorable user experience and prevent odors being matched by elicited emotions, while those with neutral or pleasant characteristics were retained. Third, the familiarity of each odor was assessed, with swiftly recognizable scents being excluded, such as rose, while odors of moderate to low familiarity (scoring below 7) were preserved for next stage. Then, odorants exhibiting unstable chemical properties (oxidation, discoloration) were excluded to guarantee that the odors would remain consistent and recognizable for at least several months. For example, limonene tends to oxidize, thus resulting in a different smell after a few months, and discoloration of vanillin could serve as a visual indicator for participants. In addition, due to the normal trigeminal function in many patients experiencing olfactory dysfunction, it was agreed by the expert panel to include a pair of trigeminal-related odorant. It should be emphasized that we conducted comprehensive comparisons among odorants with similar smells, retaining only the most suitable one for our study while removing other similar odorants.

Ethyl Maltol and propylene glycol were mixed in a ratio of 1:10, while all other odor materials were kept pure. Meanwhile, odor intensity was evaluated under the effort of 5 experts and it can be confirmed that the final odorants exhibited approximately equal intensities. We prepared 24 glass jars to contain the final 12 pairs of odorants (see Table 2). The task of the participants was to arrange them in matching pairs. Each jar was filled with 0.5 ml of the fragrance and a sling gauze pad was then placed inside to prevent loss of the liquid in the possible event of spillage. The volume of jars was 40 ml with an opening diameter of 35 mm.

**Table 2:** Demographics and descriptive statistics

	Patients (*n* = 55)	Control (*n* = 55)	F/ T/ χ^2^	*P*
Gender (M: F)	13:42	17:38	0.73	0.39
Age (years)	48.8 ± 10.5	47.0 ± 11.8	-0.85	0.39
Smoker	8	2		
Parosmia	34	0		
Phantosmia	11	0		
Diagnosis of olfactory loss				
Post-infectious	6	-		
Covid-19	38	-		
Idiopathic	6	-		
Sinonasal	1	-		
Post-traumatic	2	-		
Subjective olfactory function	3.69 ± 1.88	7.18 ± 1.38	11.11	< 0.01
Subjective nasal patency	7.37 ± 2.07	7.55 ± 1.88	0.46	0.32
Odor Identification	9.95 ± 2.42	13.75 ± 1.02	10.72	< 0.01
Odor Discrimination	10.11 ± 1.95	12.31 ± 1.63	6.42	< 0.01
Odor Threshold	4.29 ± 2.33	8.11 ± 1.98	9.27	< 0.01
TDI scores	24.34 ± 4.31	34.17 ± 2.61	14.46	< 0.01
NOST Score	4.86 ± 2.76	7.55 ± 2.64	4.80	< 0.01

### Procedure

All participants were informed about the aim, possible risks and overall outline of the study. Following written consent, the test started. Participants always had opportunities to ask questions and to quit the measurements without providing reasons.

First, participants had to complete questionnaires on medical history, their health condition and individual olfactory perception. Then, a validated and reliable olfactory test, the “Sniffin’ Sticks” test (SST) [15] was used to assess general olfactory function to divide the participants into groups with or without olfactory disorder. After a break of several minutes, the odor memory test formally started. Participants received 24 jars at once. They were allowed to unscrew the lids and then close them after smelling. Within a maximum of 15 min, the jars could be sniffed as often as needed. Participants were not allowed to make notes. All jars could be arranged or rearranged for the entire 15 min period. Each jar was labelled by a specific code at its bottom, enabling a post-test review and scoring. Patients could independently complete the entire test. Nevertheless, to ensure accuracy, all tests in the present study were conducted under the supervision and guidance of the same trained experimenter (AB).

After the odor memory test, using 11-point Likert-type scales participants were asked to answer 4 questions to assess their smell ability and performance in the tests (4 questions included). The questions related **(1)** to the participants’ confidence with their performance in the tests (0 = not satisfied at all, 10 = very satisfied), **(2)** to the ease of use (0 = very difficult to use, 10 = very easy to use), **(3)** to the effort they needed to finish the test (0 = easy, 10 = difficult), and **(4)** to the overall intensity of the odorants in the test (0 = barely perceptible, 10 = very strong).

### Statistical analysis

SPSS 29.0 was used for statistical analysis. The NOST test result was the sum of correctly matched pairs. The test validity was assessed by a comparison of NOST score between participants with and without olfactory dysfunction. Pearson correlations were used to assess test-retest reliability and correlations between NOST and SST scores. Behavioral differences between NOST and SST, and sex difference in NOST were examined by F-tests with age as a covariate. We plotted ROC curves to calculate the cutoffs of NOST scores in distinguishing hyposmia.

## Results

Table 1 showed demographics and descriptive statistics of tests scores. Half of the 110 participants were patients with olfactory dysfunction, and, expectedly, they showed significantly poorer olfactory performance than the control group, for odor identification, threshold and discrimination tests (all *p* < 0.01). This can be further indicated by the performance in each pair (see Table 3). Patients with olfactory dysfunction had significantly lower accuracy for each pair.

**Table 3:** Accuracy of the NOST items in healthy and patient groups

	Control	Patient	T	*P*
Pair 1	82%	56%	31.13	< 0.01
Pair 2	43%	36%	1.95	0.17
Pair 3	78%	64%	10.15	< 0.01
Pair 4	57%	20%	23.34	< 0.01
Pair 5	69%	52%	7.62	< 0.01
Pair 6	53%	24%	17.74	< 0.01
Pair 7	57%	32%	4.66	0.03
Pair 8	43%	32%	4.66	0.03
Pair 9	59%	28%	7.06	< 0.01
Pair 10	63%	50%	3.41	0.07
Pair 11	47%	26%	14.24	< 0.01
Pair 12	73%	44%	9.95	< 0.01

**Fig. 1:**
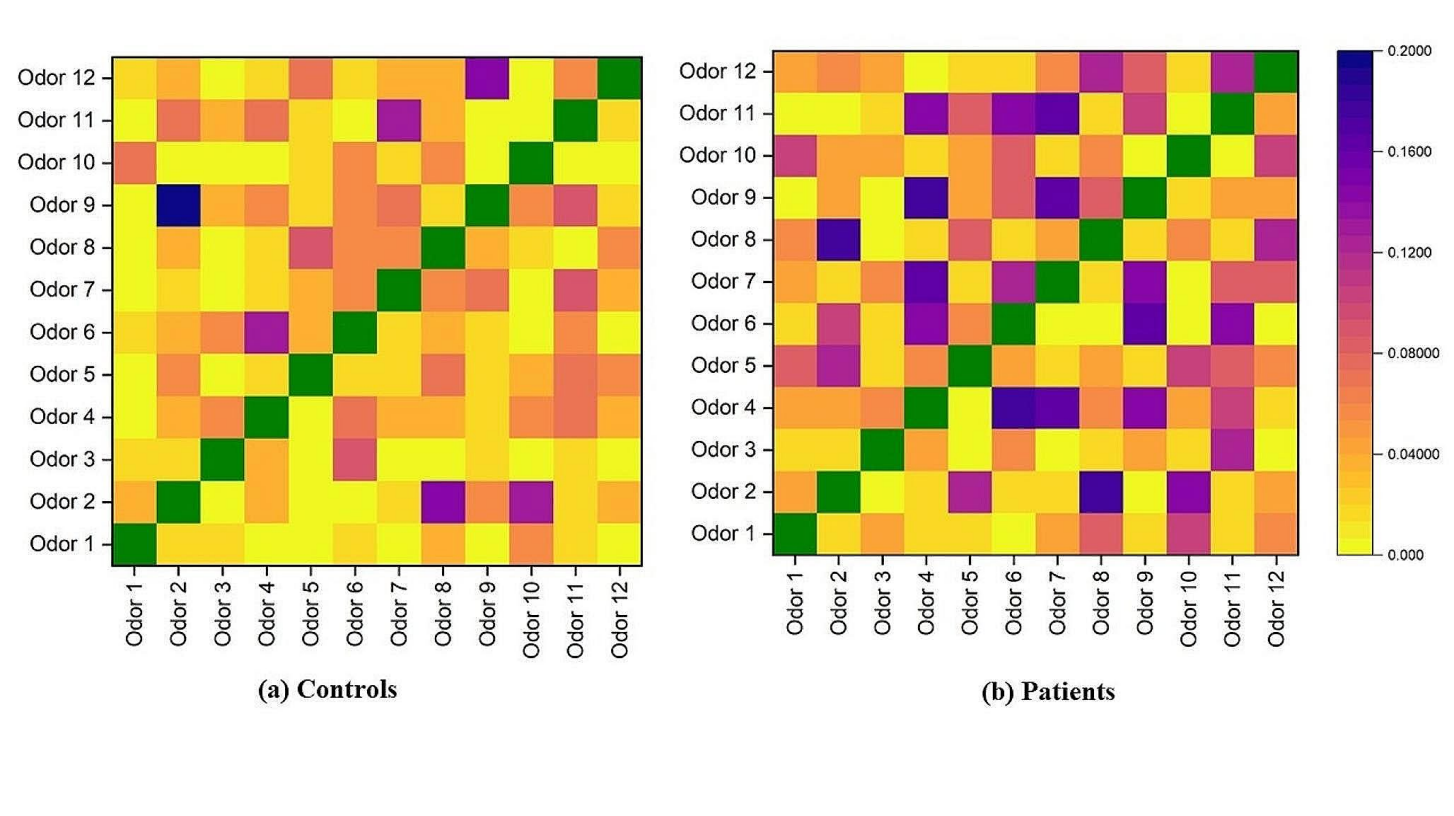
Mismatch rate of the 12 odorants. Note: Purple means a higher mismatch rate, yellow represents a lower rate, and green indicates successful matching

Moreover, there were no pairs of overly similar odors that are frequently misidentified with each other, while patients had the tendency to mismatch odor 2 with 8, odor 4 with 6, 7, 9, odor 6 with 11, and odor 7 with 9 (see Fig. 1). The level of confidence with the test and ease of use in NOST was similar to the validated SST (see Table 4). However, participants felt they had to put in a little more effort when conducting the odor memory test, and the odorants in the NOST were stronger than in the SST.

**Table 4:** Ratings of NOST and SST

	NOST	SST	T	*P*
Confidence with test	5.34 ± 2.31	5.39 ± 2.39	0.16	0.87
Ease of use	4.41 ± 2.48	4.95 ± 2.38	1.59	0.11
Efforts with the test	4.12 ± 2.91	3.57 ± 2.72	-2.30	0.02
overall odor intensity	6.04 ± 2.20	4.82 ± 2.06	-5.26	< 0.01

We found a significant age effect (*F* = 4.50, *p* = 0.04) but no gender effect even with age as a covariate (*F* = 0.02, *p* = 0.90). Also, no interaction between factors “age” and “gender” was observed (*F* = 0.38, *p* = 0.54, see Table 5).

**Table 5:** Age and gender effect

	NOST score	F	*P*
Gender		0.02	0.90
Men (*n* = 25)	7.32 ± 3.16		
Women (*n* = 68)	5.97 ± 2.88		
Age		4.50	0.04
Age * Gender		0.38	0.54

**Fig. 2:**
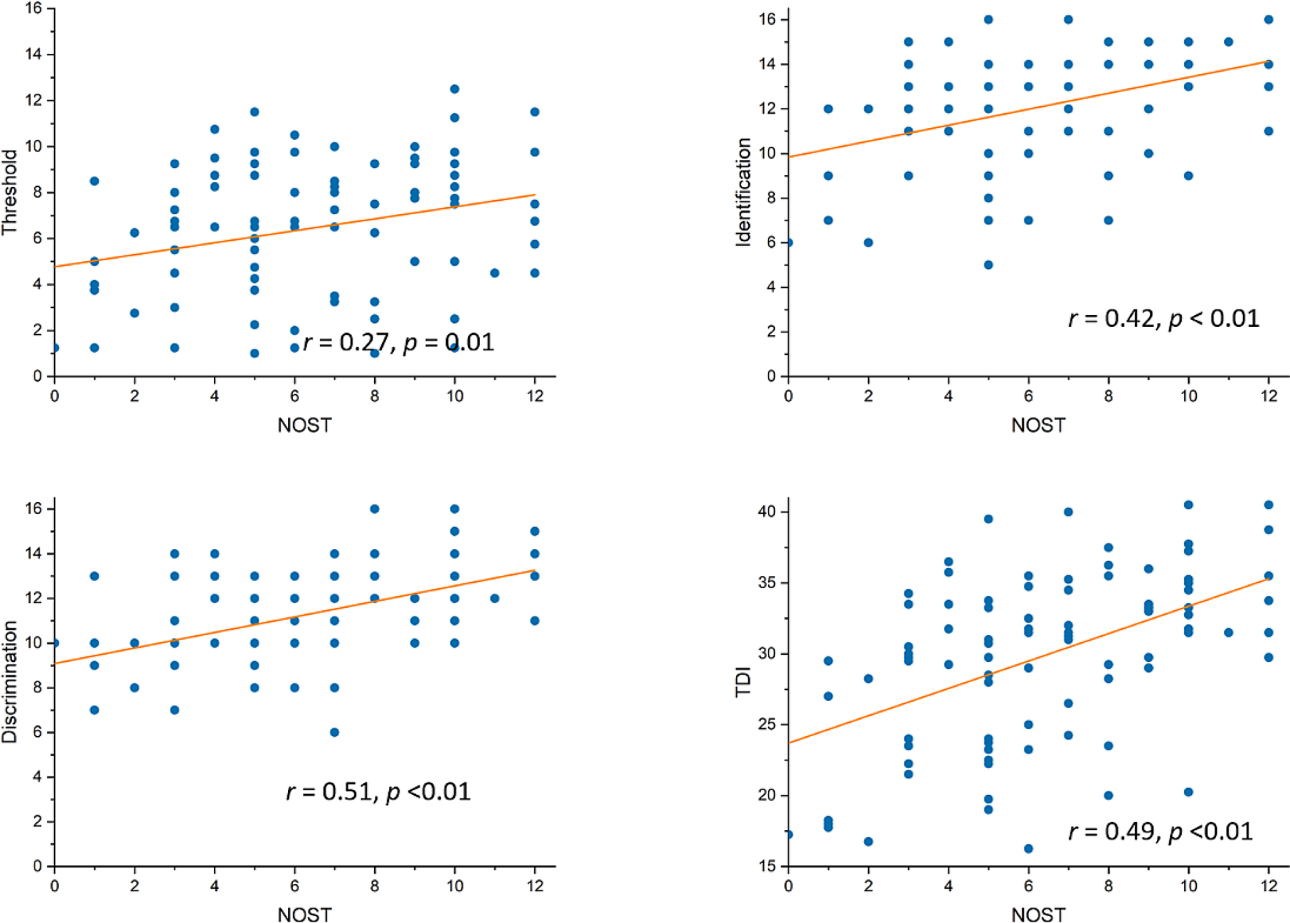
Correlations between NOST and subtests of SST

The NOST score showed low but significant correlations with the SST (threshold test: *r* = 0.27, *p* = 0.01; discrimination test: *r* = 0.51, *p* < 0.01; identification test: *r* = 0.42, *p* < 0.01; TDI scores: *r* = 0.49, *p* < 0.01, Fig. 2). Hyposmic patients showed worse performance in NOST than normosmic individuals even after introducing age as a covariate (Controls = 7.55 ± 2.64, Patients: 4.86 ± 2.76, *F* = 23.32, *p* < 0.01), suggesting its applicability for differentiating hyposmia from normosmia.

**Fig. 3:**
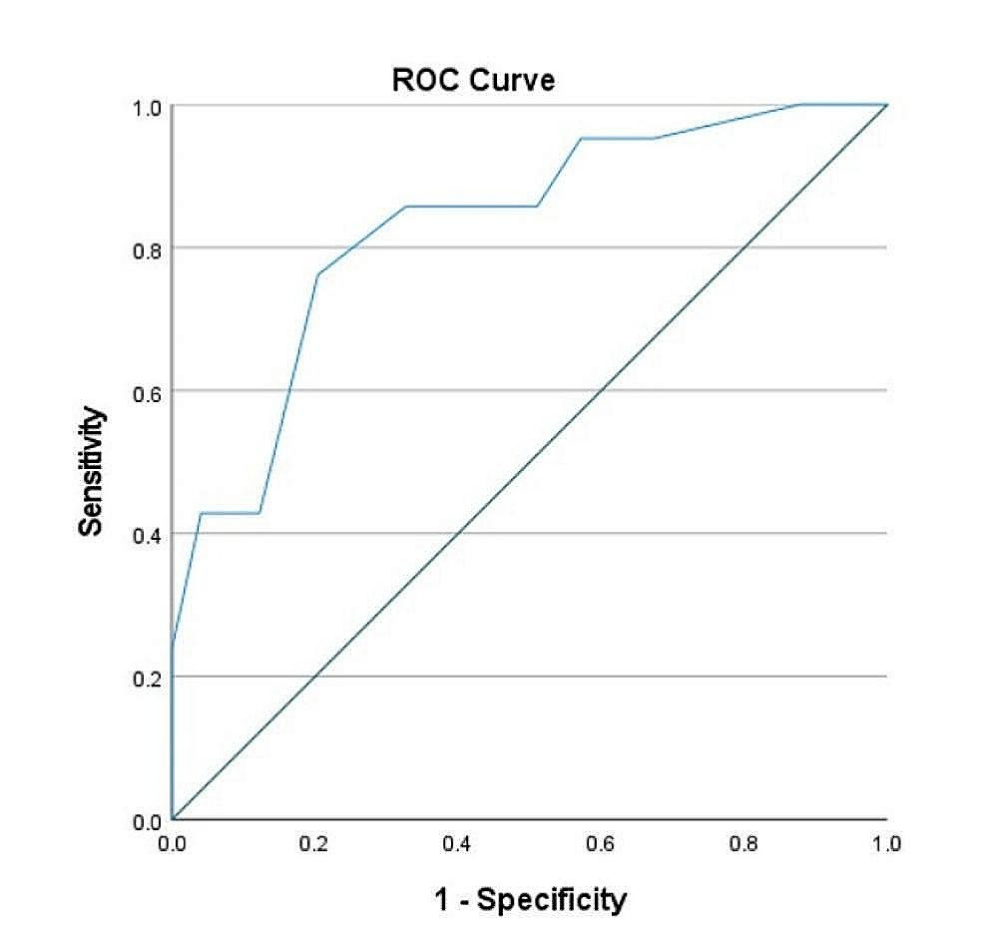
ROC curve of NOST

The ROC analysis revealed an area under the curve (AUC) of 0.815 (SE = 0.055, asymptotic significance < 0.001, asymptotic 95% CI = 0.707 to 0.924). The NOST score with the maximal Youden’s index was 5.5, leading to a sensitivity of 76.2%, and specificity of 77.6% of detecting severe hyposmic patients (TDI < 24) from normosmic people (TDI > 31) (Fig. 3; Table 6).

**Table 6:** ROC analysis of NOST score to detect hyposmia

Hyposmia if NOST score ≤	Sensitivity	Specificity	Youden’s index
0.5	0.048	1.000	0.048
1.5	0.190	1.000	0.190
2.5	0.238	1.000	0.238
3.5	0.429	0.939	0.367
4.5	0.429	0.857	0.286
**5.5**	**0.762**	**0.776**	**0.537**
6.5	0.857	0.653	0.510
7.5	0.857	0.469	0.327
8.5	0.952	0.408	0.361
9.5	0.952	0.327	0.279
10.5	1.000	0.122	0.122
11.5	1.000	0.102	0.102
13	1.000	0.000	0.000

Note: Bold value indicate the maximal Youden’s index

## Discussion

Despite various possibilities to assess odor memory, currently none of the available tests can be performed by individuals themselves in an unassisted manner. Hence, this study aimed to develop an objective, self-administered assessment based on an odor memory task. To make it easy to use and comprehend, we conducted a memory match game, with 12 pairs needed to be matched from 24 odors.

A relatively stable test performance was observed after 14 days with a significant correlation coefficient of 0.45. However, the retest reliability is not as ideal as expected, which may result from the limited number of items (12 pairs of odorants). It has been reported that the coefficients of correlations between test and retest scores decreased from 0.93 over 0.73 to 0.60 when reducing the number of olfactory items from 32 over 16 to 12 (e.g., [16, 17]). In addition, it has to be kept in mind that the subgroup invited for the test-retest analysis were relatively homogeneous in terms of their olfactory function. If this variance between tested individuals had been larger it is conceivable that the test-retest reliability would have increased. The self-ratings of NOST and SST showed that, compared with the SST, participants could properly finish the NOST, and were satisfied with their own test performance.

The present NOST showed a good validity relating to the SST, a standardized clinical test. It is well-established that olfactory impairments in neurodegenerative diseases can be detected through olfactory identification, odor sensitivity and other dimensions of olfactory function [18]. Cognitive deficits in memory loss, dementia, are important early signs of prodromal neurodegenerative disease [19]. Therefore, an olfactory memory test, a combined index of olfactory and cognitive function, could be a promising method for the diagnosis for neurodegenerative disease [20].

As a home test, the NOST presents some advantages that are not satisfied by traditional olfactory tests. First, it can be conducted by individuals themselves so that patients can establish their olfactory disorders quantitatively without the immediate need to present themselves repetitively in specialized centers – these may be of specific significance in rural areas with some distance to larger cities, or also in clinics with no established smell and taste dysfunction department. Having said that, however, the test is not meant to replace professional diagnosis and counseling.

Home tests are often available for online purchase or pharmacies, making them accessible to a wide range of people. It removes the necessity of making appointments and waiting in clinics, thereby saving patients’ time. Besides, it seems to be useful for the rehabilitation of patients with olfactory dysfunction. Due to the widespread olfactory dysfunction caused by COVID-19, the NOST can serve as a quick, convenient home test that allows the patient to track olfactory function (which is different form olfactory screening tests based on odor identification where the correct odors are quickly learned and memorized which is a strong bias for consecutive tests). Furthermore, the frequent odor exposure that the NOST may provide, appears to make it a perfect companion for olfactory training [21]. With the present test, individuals could be able to take control of their health of olfactory function by providing them with tools to monitor and manage their well-being independently. This could be another promising direction for the future research and application.

What is more, the NOST revealed a poorer performance of olfactory function in severely hyposmic patients compared to the normosmic group, further suggesting its effect to differentiate between severe hyposmia and normosmia. Hyposmia can be predicted by the present test when using a score of 5.5 with a sensitivity of 76.2% and specificity of 77.6%.

In accordance with the previous results, the present results suggest that older people have worse performance in NOST, suggesting a significant age-related olfactory and cognitive decline [22, 23]. As for the gender effect of olfactory functions, many studies reported that women outperform men [11, 12, 14], while some found no significant differences [24]. These somewhat controversial results can be explained by the weak effect size for the factor “gender” ranging from 0.08 to 0.30 [25]. In fact, for the current study, no gender effect was found.

Despite of these promising results, several limitations remain. First, the current test was performed with small glass containers, which are very practical but bulky. Because of that, the test may not be very convenient to use when space is limited. The NOST could be improved for self-administration if it was presented in a more portable and convenient form, like smaller bottles or other delivery devices. Furthermore, the NOST should also be applied in anosmic patients in future studies to evaluate whether it can distinguish anosmia from hyposmia/normosmia. In addition, as an auxiliary diagnostic tool, the selection of norms and cutoff values for the NOST is crucial, which is expecting a more accurate result by the future studies with larger sample sizes.

In conclusion, the present study showed the good reliability, validity and possible clinical usage of the NOST. Compared with existing tools, it can be comprehended and conducted easily, and without any help from others, which may provide a quick and simple approach to get a global estimation of one’s olfactory and cognitive condition. Among others it may help not only to facilitate the early diagnosis of neurodegenerative diseases, but also to recognize olfactory dysfunction as well as recovery from olfactory loss.

## Electronic supplementary material

Below is the link to the electronic supplementary material.

Supplementary Material 1

## References

[1] Desiato VM, Levy DA, Byun YJ, Nguyen SA, Soler ZM, Schlosser RJ (2021) The prevalence of olfactory dysfunction in the General Population: a systematic review and Meta-analysis. Am J Rhinol Allergy 35(2):195–205. 10.1177/1945892420946254 32746612 10.1177/1945892420946254 PMC13080788

[2] Yang J, Pinto JM (2016) The epidemiology of olfactory disorders. Curr Otorhinolaryngol Rep 4(2):130–141. 10.1007/s40136-016-0120-6 30613439 10.1007/s40136-016-0120-6 PMC6317880

[3] Vaira LA, Salzano G, Le Bon SD, Maglio A, Petrocelli M, Steffens Y, Ligas E, Maglitto F, Lechien JR, Saussez S, Vatrella A, Salzano FA, Boscolo-Rizzo P, Hopkins C, De Riu G (2022) Prevalence of persistent olfactory disorders in patients with COVID‐19: a psychophysical case‐control study with 1‐Year follow‐up. Otolaryngology–Head Neck Surg 167(1):183–186. 10.1177/01945998211061511 10.1177/01945998211061511 34813382

[4] Wehling E, Nordin S, Espeseth T, Reinvang I, Lundervold AJ (2011) Unawareness of olfactory dysfunction and its Association with Cognitive Functioning in Middle aged and old adults. Arch Clin Neuropsychol 26(3):260–269. 10.1093/arclin/acr019 21474482 10.1093/arclin/acr019

[5] Karstensen H, Tommerup N (2012) Isolated and syndromic forms of congenital anosmia. Clin Genet 81(3):210–215. 10.1111/j.1399-0004.2011.01776.x 21895637 10.1111/j.1399-0004.2011.01776.x

[6] Oleszkiewicz A, Hummel T (2019) Whose nose does not know? Demographical characterization of people unaware of anosmia. Eur Arch Otorhinolaryngol 276(6):1849–1852. 10.1007/s00405-019-05414-8 30989334 10.1007/s00405-019-05414-8 PMC6529373

[7] Doty RL (2022) Olfactory dysfunction in COVID-19: Pathology and long-term implications for brain health. Trends Mol Med 10.1016/j.molmed.2022.06.005 PMC9212891 35810128

[8] Knaapila A, Tuorila H, Kyvik KO, Wright MJ, Keskitalo K, Hansen J, Kaprio J, Perola M, Silventoinen K (2008) Self-ratings of olfactory function reflect odor annoyance rather than olfactory acuity. Laryngoscope 118(12):2212–2217 18948833 10.1097/MLG.0b013e3181826e43

[9] Li Z, Stolper S, Draf J, Haehner A, Hummel T (2022) Smell, taste and trigeminal function: similarities and differences between results from home tests and examinations in the clinic. Rhinology 60(4):293–300 35926120 10.4193/Rhin21.430

[10] Herz RS, Engen T (1996) Odor memory: review and analysis. Psychon Bull Rev 3(3):300–313. 10.3758/BF03210754 24213931 10.3758/BF03210754

[11] Croy I, Zehner C, Larsson M, Zucco GM, Hummel T (2015) Test-retest reliability and validity of the Sniffin’ TOM odor memory test. Chem Senses 40(3):173–179. 10.1093/chemse/bju069 25550307 10.1093/chemse/bju069

[12] Sorokowska A, Sabiniewicz A, Larsson M (2020) TOM-32–An extended test for the assessment of olfactory memory. J Neurosci Methods 344:108873 32710924 10.1016/j.jneumeth.2020.108873

[13] Li S, Yan C, Hummel T, Zou L (2023) Development and validation of the olfactory memory test battery (OMTB) based on odors with high- and low-verbalizability. J Neurosci Methods 388:109826. 10.1016/j.jneumeth.2023.109826 36822275 10.1016/j.jneumeth.2023.109826

[14] Choudhury ES, Moberg P, Doty RL (2003) Influences of age and sex on a microencapsulated odor memory test. Chem Senses 28(9):799–805 14654448 10.1093/chemse/bjg072

[15] Hummel T, Sekinger B, Wolf SR, Pauli E, Kobal G (1997) Sniffin’sticks’: olfactory performance assessed by the combined testing of odor identification, odor discrimination and olfactory threshold. Chem Senses 22(1):39–52 9056084 10.1093/chemse/22.1.39

[16] Doty RL, McKeown DA, Lee WW, Shaman P (1995) A study of the test-retest reliability of ten olfactory tests. Chem Senses 20(6):645–656. 10.1093/chemse/20.6.645 8788098 10.1093/chemse/20.6.645

[17] Haehner A, Mayer A-M, Landis BN, Pournaras I, Lill K, Gudziol V, Hummel T (2009) High test-retest reliability of the Extended Version of the Sniffin’ sticks Test. Chem Senses 34(8):705–711. 10.1093/chemse/bjp057 19759361 10.1093/chemse/bjp057

[18] Fatuzzo I, Niccolini GF, Zoccali F, Cavalcanti L, Bellizzi MG, Riccardi G, De Vincentiis M, Fiore M, Petrella C, Minni A, Barbato C (2023) Neurons, nose, and neurodegenerative diseases: olfactory function and cognitive impairment. Int J Mol Sci 24(3):2117. 10.3390/ijms24032117 36768440 10.3390/ijms24032117 PMC9916823

[19] Bottiroli S, Bernini S, Cavallini E, Sinforiani E, Zucchella C, Pazzi S, Cristiani P, Vecchi T, Tost D, Sandrini G& others. (2021) The smart aging platform for assessing early phases of cognitive impairment in patients with neurodegenerative diseases. Front Psychol 12:635410 33790839 10.3389/fpsyg.2021.635410 PMC8005545

[20] Bahuleyan B (2012) Olfactory memory impairment in neurodegenerative diseases. J Clin Diagn Res. 10.7860/JCDR/2012/3408.2382 23205370 10.7860/JCDR/2012/3408.2382 PMC3471510

[21] Kattar N, Do TM, Unis GD, Migneron MR, Thomas AJ, McCoul ED (2021) Olfactory training for Postviral olfactory dysfunction: systematic review and Meta-analysis. Otolaryngology–Head Neck Surg 164(2):244–254. 10.1177/0194599820943550 10.1177/0194599820943550 32660334

[22] Doty RL, Kamath V (2014) The influences of age on olfaction: a review. Front Psychol 5:20 24570664 10.3389/fpsyg.2014.00020 PMC3916729

[23] Ferreira N, Owen A, Mohan A, Corbett A, Ballard C (2015) Associations between cognitively stimulating leisure activities, cognitive function and age-related cognitive decline. Int J Geriatr Psychiatry 30(4):422–430 24989949 10.1002/gps.4155

[24] Sorokowska A, Schriever VA, Gudziol V, Hummel C, Hähner A, Iannilli E, Sinding C, Aziz M, Seo H, Negoias S (2015) & others. Changes of olfactory abilities in relation to age: Odor identification in more than 1400 people aged 4 to 80 years. *European Archives of Oto-Rhino-Laryngology*, *272*, 1937–1944 10.1007/s00405-014-3263-4 PMC4473282 25238811

[25] Sorokowski P, Karwowski M, Misiak M, Marczak MK, Dziekan M, Hummel T, Sorokowska A (2019) Sex differences in human olfaction: a Meta-analysis. Front Psychol 10:242. 10.3389/fpsyg.2019.00242 30814965 10.3389/fpsyg.2019.00242 PMC6381007

