# Correction: A novel olfactory sorting task

**DOI:** 10.1007/s00405-024-08909-1

**Published:** 2024-10-07

**Authors:** Shubin Li, Anne Wolter, Christine Kelly, Barry Smith, Katie Whitcroft, Harry Sherwood, Beth Longley, Thomas Hummel

**Affiliations:** 1 Department of Otorhinolaryngology, Smell & Taste Clinic, Technische Universität Dresden, Dresden, Germany; 2 AbScent, 14 London Street, Andover, Hampshire, UK; 3 Institute of Philosophy, School of Advanced Study, University of London, London, UK; 4 Centre for Olfactory Research and Applications, School of Advanced Study, University of London, London, UK; 5 Institute of Cognitive Neuroscience, UCL, London, UK

**Correction: European Archives of Oto-Rhino-Laryngology**

10.1007/s00405-024-08811-w

In this article the author names Shubin Li, Anne Wolter, Christine Kelly, Barry Smith, Katie Whitcroft, Harry Sherwood, Beth Longley and Thomas Hummel were incorrectly written as Li Shubin, Becker Anne, Kelly Christine, Smith Barry, Whitcroft Katie, Sherwood Harry, Longley Beth and Hummel Thomas.

The original article has been corrected.

