# Supplementary material for: A novel olfactory sorting task
Source: Eur Arch Otorhinolaryngol. 2024 Jul 13;281(11):5823–30. doi: 10.1007/s00405-024-08811-w (PMC11512826; doi:10.1007/s00405-024-08811-w)
Supplement: Supplementary file 1 — Supplementary Material 1 [file 405_2024_8811_MOESM1_ESM.docx]

|  | CAS number | Name |
| --- | --- | --- |
| 1 | 10461-98-0 | Peonile |
| 2 | 94201-19-1 | Methyl Laitone |
| 3 | 68991-97-9 | Melafleur |
| 4 | 31906-04-4 | Lyral |
| 5 | 52474-60-9 | Precyclemone B |
| 6 | 27606-09-3 | Magnolan |
| 7 | 16409-43-1 | Rose Oxide |
| 8 | 71077-31-1 | Floral Super |
| 9 | 8006-81-3 | Ylang Ylang oil |
| 10 | 121-33-5 | Vanillin |
| 11 | 2563-07-7 | Ultra vanil |
| 12 | 928-96-1 | Cis-3-Hexanol |
| 13 | 68039-49-6 | Triplal |
| 14 | 1335-66-6 | Iso Cyclo citral |
| 15 | 81782-77-6 | Undecavertol |
| 16 | 32388-55-9 | Vertofix coeur |
| 17 | 77-54-3 | Cedryl acetate |
| 18 | 36306-87-3 | Kephalis |
| 19 | 476332-65-7 | Amber Xtreme |
| 20 | 122760-84-3 | Prismantol |
| 21 | 112-31-2 | Aldehyde c-10 |
| 22 | 2437-25-4 | Clonal |
| 23 | 141773-73-1 | Helvetolide |
| 24 | 119-53-9 | Benzoin |
| 25 | 88-40-4 | 2-tert-butyl-5-methyl anisole |
| 26 | 106-44-5 | Para Cresol |
| 27 | 104-21-2 | Anisyl Acetate |
| 28 | 15848-49-4 | Sultanene |
| 29 | 16587-71-6 | Orivone |
| 30 | 562-74-3 | Terpinen-4-ol |
| 31 | 127-41-3 | Alpha-Ionone |
| 32 | 6931-54-0 | Rosemarel |
| 33 | 7549-37-3 | Citral Dimethyl Acetal |
| 34 | 2705-87-5 | Allyl-Cyclohexyl Propionate |
| 35 | 13816-33-6 | Cumin Nitrile |
| 36 | 4940-11-8 | Ethyl Maltol |

**Supplementary**

**Table. The pool of odorants**
